# 1ppm-detectable hydrogen gas sensors by using highly sensitive P+/N+ single-crystalline silicon thermopiles

**DOI:** 10.1038/s41378-023-00506-2

**Published:** 2023-03-20

**Authors:** Haozhi Zhang, Hao Jia, Zao Ni, Ming Li, Ying Chen, Pengcheng Xu, Xinxin Li

**Affiliations:** 1 State Key Laboratory of Transducer Technology, Shanghai Institute of Microsystem and Information Technology, Chinese Academy of Sciences, 200050 Shanghai, China; 2 School of Microelectronics, University of Chinese Academy of Sciences, 100049 Beijing, China

**Keywords:** Electrical and electronic engineering, Chemistry

## Abstract

Hydrogen (H_2_) is currently of strategic importance in the pursuit of a decarbonized, environmentally benign, sustainable global energy system; however, the explosive nature of H_2_ requires leakage monitoring to ensure safe application in industry. Therefore, H_2_ gas sensors with a high sensitivity and fast response across a wide concentration range are crucial yet technically challenging. In this work, we demonstrate a new type of MEMS differential thermopile gas sensor for the highly sensitive, rapid detection of trace H_2_ gas in air. Facilitated by a unique MIS fabrication technique, pairs of single-crystalline silicon thermopiles (i.e., sensing and reference thermopiles) are batch fabricated with high-density single-crystalline silicon thermocouples, yielding an outstanding temperature sensitivity at the sub-mK level. Such devices ensure the detection of miniscule temperature changes due to the catalytic reaction of H_2_ with a detection limit as low as ~1 ppm at an operating temperature of 120 °C. The MEMS differential thermopiles also exhibit a wide linear detection range (1 ppm-2%, more than four orders of magnitude) and fast response and recovery times of 1.9 s and 1.4 s, respectively, when detecting 0.1% H_2_ in air. Moreover, the sensors show good selectivity against common combustible gases and volatile organics, good repeatability, and long-term stability. The proposed MEMS thermopile H_2_ sensors hold promise for the trace detection and early warning of H_2_ leakage in a wide range of applications.

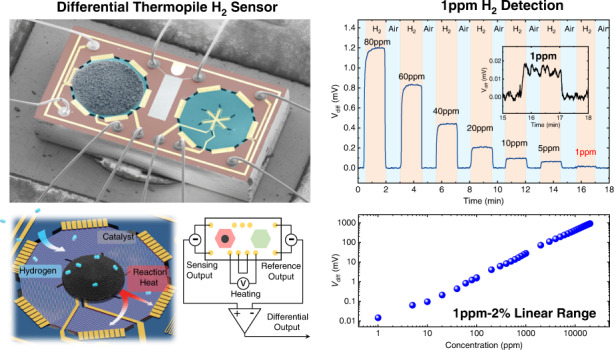

## Introduction

Hydrogen (H_2_) is regarded as one of the most promising green energies due to its advantages of cleanliness, efficiency and sustainability. Moreover, hydrogen gas is also widely used in aerospace, petrochemical engineering and many other fields. However, due to the wide concentration range in which it can explode (4–75% in air) and low ignition energy (0.02 mJ), hydrogen gas is highly explosive and dangerous during production, storage and transportation^1^. To improve the safety of hydrogen energy applications, the leakage risk of hydrogen gas needs to be reliably monitored. Developing high-performance hydrogen gas sensors with low-concentration detection limits, wide measurement ranges, and fast responses is highly desirable^2^.

Hydrogen (H_2_) gas sensors have been intensively studied for decades. Various types of hydrogen sensors, including metal oxide semiconductor (MOS) sensors^3–6^, electrochemical sensors^7^, work function-based sensors (e.g., Schottky diode sensors, FET sensors)^8–11^, and catalytic reaction sensors^12^, have been developed, and many efforts have been made to improve hydrogen gas detection performance. Generally, oxide semiconductor sensors and electrochemical sensors have high sensitivity, and low concentrations of hydrogen gas can be detected^3,13–18^. However, the applications of these sensors are restricted due to their narrow detection ranges (1–2 orders of magnitude) and slow responses (several seconds up to hundreds of seconds)^17,19^. In contrast, catalytic reaction-based H_2_ sensors are promising for flammable gas detection due to their broader detection range. Traditional catalytic reaction sensors (namely, “pellistors”), which consist of alumina beads loaded with a catalyst and a platinum thermistor for heating and temperature measurement^20,21^, have been hindered by their slow response time (>60 s), detection limit (~0.1%) and high power consumption (hundreds of mW)^22^. With the rapid development of MEMS technology, pellistor sensors have been developed into miniaturized thermoelectric detection devices, with a significant improvement in both power consumption and sensitivity^23–27^. By constructing an insulated cavity and a MEMS thermoelectric generator, a developed SiGe-based hydrogen sensor had an extended limit of detection of 50 ppm or lower^25,28^.

As an alternative type of thermoelectric device, MEMS thermopiles have recently received significant attention in gas sensing applications due to their high sensitivity, high signal-to-noise ratio and low power consumption^29–31^. To date, polysilicon thermopiles loaded with platinum (Pt) catalysts have been typically reported for H_2_ sensing, with a detection limit of ~10 ppm and a detection range of ~10 ppm-1.5% (more than three orders of magnitude)^32,33^. However, these characteristics are still not sufficient, as trace hydrogen at an even lower concentration of approximately 1 ppm needs to be detected in many hydrogen gas leakage detection applications.

Thermoelectric hydrogen gas sensors measure the generated heat from the oxidization of hydrogen gas, so the major factors that affect the detection limit are catalysis efficiency and sensitivity to the heat-induced temperature change in the sensing structure. By improving the catalyst efficiency, several works achieved a detection level of ~10 ppm by optimizing the catalyst^26,32–35^. In terms of the thermopile temperature sensing element, the reported works have generally used polysilicon thermocouples. The equivalent Seebeck coefficient, i.e., the product of the Seebeck coefficient and the thermocouple number of the thermopile, reflects the temperature detection sensitivity of the thermoelectric device. Due to the relatively low Seebeck coefficient of polysilicon^36^, polysilicon-based devices have not been reported for the detection of hydrogen gas at levels <10 ppm. To significantly lower the detection limit of thermoelectric hydrogen gas sensors to 1 ppm, it is necessary to replace the polysilicon thermoelectric material with a new material with a much higher Seebeck coefficient, e.g., single-crystalline silicon. It is well known that single-crystalline silicon has a Seebeck coefficient several times higher than that of polysilicon^37^. Unfortunately, normally, thermopile material has to be deposited on top of a thermal insulating thin film (e.g., suspended silicon nitride), and the deposition of single-crystalline silicon thermopile seems hard to be made on top of the insulating thin film. This may be the reason why single-crystalline silicon has rarely been reported as a thermopile material in previous works.

In this work, we develop a highly sensitive differential thermopile hydrogen gas sensor with MEMS technologies. The sensor consists of two identical, heating-enabled thermopiles, with each thermopile consisting of 54 pairs of thermocouples. More importantly, the thermocouple material is single-crystalline silicon instead of polysilicon. Due to the much higher Seebeck coefficient of single-crystalline silicon, the proposed hydrogen sensors achieve a greatly improved detection limit of 1 ppm. With the Pt NPs@Al_2_O_3_ catalyst loaded on the sensing thermopile for hydrogen gas detection, the sensors demonstrate excellent selectivity, uniformity, and long-term stability, thereby holding promise for various hydrogen gas detection applications.

## Results

### Sensor design and fabrication

**Fig. 1:**
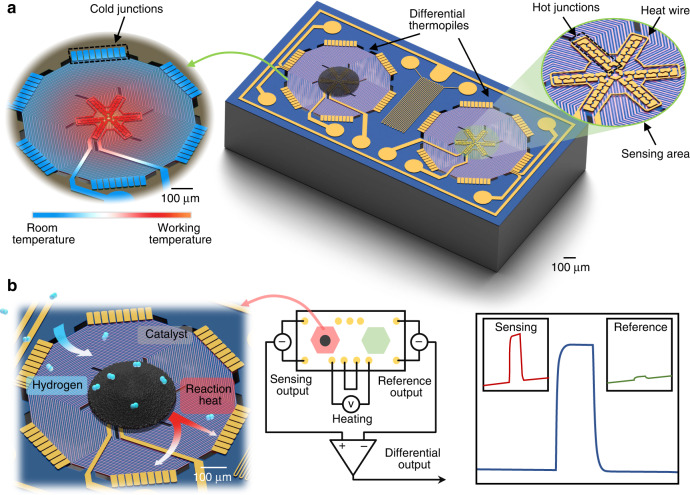
Structural design and working principle of the MEMS differential thermopile H_2_ sensor. **a** Schematic diagram of the MEMS differential thermopile-based H_2_ sensor, consisting of a sensing thermopile and a reference thermopile. **b** Schematic diagram of the H_2_ sensing mechanism and working principle of the sensor. The differential output signal is proportional to the temperature change due to the H_2_ catalytic reaction process, while environmental interferences are subtracted

As illustrated in Fig. 1a, the sensor is composed of MEMS differential thermopiles, with two identical thermopiles suspended on a thermally insulating diaphragm and a heating voltage applied to control the working temperature. The left thermopile for heat sensing is coated with the catalyst for selective reaction with hydrogen gas. To eliminate the influence of environmental factors, such as the thermal conductivity and flow rate of the gas, the thermopile on the right side is designed for reference and compensation. The thermopile has an area with a diameter of 640 µm and consists of 54 pairs of single-crystalline silicon thermocouples in series. Fifty-four N-type thermocouple beams and 54 P-type thermocouple beams are arranged alternately, and each pair of adjacent N-type/P-type thermocouple beams (N-type on the left and P-type on the right) are directly connected at the hot end. At the cold end, they are connected to another adjacent thermocouple pair. The single-crystalline silicon thermopile is suspended at the backside of a low-stress silicon nitride adiabatic support membrane and thermally isolated from the silicon substrate via an etched air cavity. The hot junctions of the thermocouples are uniformly distributed in a 240 μm-diameter region at the center of the suspended membrane, which is called the sensing region. Moreover, the cold junctions are connected in parallel to a silicon frame heat sink. A single thermocouple can detect the temperature difference between the hot junction and the cold junction so that this thermopile detects the average temperature difference between the sensing region and the environment. At each thermopile area, a heating resistor pattern around the sensing region is designed for heating the membrane to the desired temperature for the catalyst. Because of the suspended membrane structure of the thermopile, the heat is confined to the vicinity of the heating resistor, thereby significantly reducing the power consumption of the sensor. In between the two thermopiles, a Pt thermistor with a serpentine pattern is designed to detect the ambient temperature. In addition, a metal guard ring around the entire device is also designed for noise shielding.

Figure 1b illustrates the operation of the sensor. The catalytic sensing material is loaded in the sensing region. The sensing regions of both thermopiles are heated to the operating temperature by supplying the same heating voltage. At the operating temperature, the catalyst dissociates H_2_ into H atoms on the surface^5,38^ and generates surface hydroxyl OH groups with dissociated O_2_. The surface hydroxyl groups are further oxidized to produce water^39^. The reaction of H_2_ and O_2_ releases heat, thereby increasing the output voltage of the sensing thermopile^40^. In the meantime, the reference thermopile only responds to the original temperature and the common-mode disturbance caused by environmental change. The differential output of the two thermopiles reflects the specific signal induced by the selectively oxidized hydrogen gas.

According to the Seebeck effect of the thermocouple, the differential output voltage V_diff_ of the sensor is expressed as

1 $$V_{diff} = V_{sensing} - V_{reference} = N(\alpha _1 - \alpha _2)\Delta T_{h2}$$

where $V_{sensing} = N\left( {\alpha _1 - \alpha _2} \right)\left( {T_{heat} + \Delta T_{h2} + \Delta T_{cm} - T_{env}} \right)$ and $V_{reference} = N(\alpha _1 - \alpha _2)(T_{heat} + \Delta T_{cm} - T_{env})$. N is the number of thermocouple pairs, α_1_ and α_2_ are the Seebeck coefficient values of the two materials to form the hot junctions, T_heat_ is the temperature generated by the heater, ΔT_h2_ is the temperature increase generated by hydrogen oxidation, ΔT_cm_ is the temperature change caused by common-mode interference, and T_env_ is the cold junction temperature, which equals the ambient temperature. The temperature change caused by common-mode interference is not negligible. Since the thermal conductivity of hydrogen gas is significantly higher than that of air, the sensor loses more heat in the hydrogen atmosphere and cause a temperature change. This is a nonspecific response to the change in gas thermal conductivity. In addition, gas flow rate variation and other factors also produce common-mode interference. Therefore, the reference thermopile for eliminating the common-mode interference is very important in the sensor.

To improve the sensitivity of the thermopile, single-crystalline silicon is selected as the thermoelectric material for constructing the thermocouples. The Seebeck coefficient of single-crystalline silicon is approximately three times that of polysilicon^36,37^. By using the N-type/P-type single-crystalline silicon thermocouple structure, the Seebeck coefficient of the thermocouple is 6 times that of the traditional N-type polysilicon/metal thermocouple. Because of the low thermal conductivity of the silicon nitride film^41^, the heat is conducted primarily through the single-crystalline silicon thermocouple beams. Therefore, the thermocouples are optimized into a spiral shape to reduce heat transfer.

Quite different from the reported thermopiles that are routinely fabricated on the surface of dielectric films using polysilicon deposition and backside etching techniques, we take a unique approach to fabricating MEMS thermopiles using the “microholes interetch and sealing” (MIS) micromachining technique based on a (111) silicon wafer^42,43^. Conventional (100) wafers are rarely used to fabricate single-crystalline silicon thermocouple structures with specific doping concentrations and are typically used to fabricate polycrystalline silicon thermopiles. On the other hand, although SOI wafers can be used to fabricate single-crystalline silicon thermocouple structures, the thickness of the single-crystalline silicon thermocouples is defined by the device layer. Such a process has the problems of a nonuniform thickness over the entire wafer scale and the need for double-sided alignment to release the device from the backside. In the MIS process, the single-crystalline silicon thermocouples are fabricated from (111) silicon, while the thickness of the single-crystalline silicon thermocouples and the depth of the thermal insulation cavity are defined by the depth of the RIE etch, which has good uniformity. Moreover, the designed etch holes and (111) wafers allow us to create the thermal insulation cavity just from the front side without double-sided processing. MIS technology allows the building of complex single-crystalline silicon MEMS structures with a cost-effective, single-sided process using non-SOI wafers, hence greatly improving the uniformity (e.g., device thickness) and lowering the cost of batch-fabricated MEMS thermopile sensors.

**Fig. 2:**
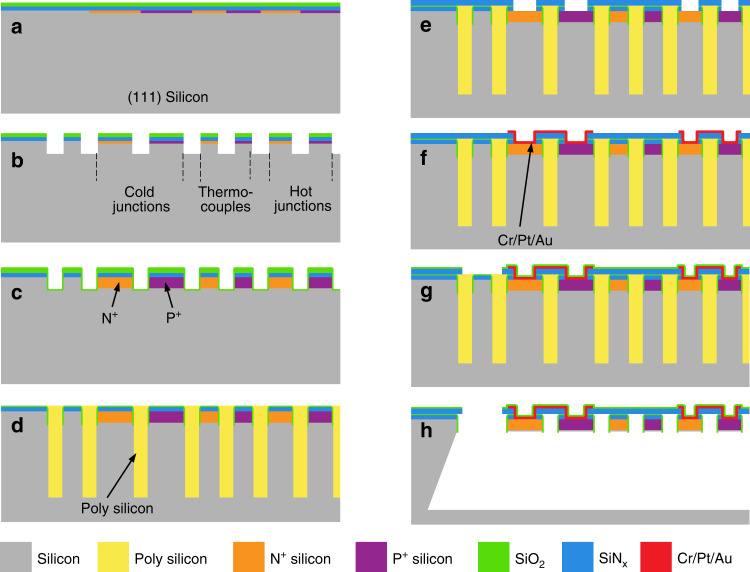
Fabrication process of the MEMS differential thermopile-based H_2_ sensor. **a** Ion implantation and dielectric film deposition. **b** Thermocouple patterning and thickness definition. **c** Diffusion and DRIE etching. **d** Polysilicon deposition and self-stopping polishing. **e** SiN_x_ deposition and contact hole etching. **f** Metal sputtering and electrode patterning. **g** SiO_2_ deposition and release hole etching. **h** TMAH etching

As illustrated in Fig. 2, the fabrication steps of MEMS thermopile devices start with a single-side-polished n-type 4-in (111) wafer. Boron and phosphorus ions are implanted at ~50 keV with doses of 8 × 10^15^ and 1 × 10^16^ ion/cm^2^, respectively. Then, low-pressure chemical vapor deposited (LPCVD) SiN_x_/SiO_2_ layers with thicknesses of 0.5 μm and 0.4 μm are patterned to form p-type and n-type thermocouple regions, respectively (Fig. 2a). Subsequently, the thickness of the single-crystalline silicon thermocouple is defined by a 4 μm-deep reactive ion etching (DRIE) (Fig. 2b). A 0.4 μm-thick LPCVD SiO_2_ layer is then anisotropically etched to form the sidewall structures, which protect the thermocouple beams against the final step of wet etching. A 40 μm-deep DRIE defines the depth of the thermally isolated cavity (Fig. 2c). Then, polysilicon is deposited to fill the deep trenches, and the surfaces are smoothed using chemical mechanical polishing (CMP) (Fig. 2d). CMP uses a polishing solution with high selectivity toward polysilicon against SiO_2_; hence, self-stopping polishing is realized when the SiO_2_ layer is exposed. After a 1 µm-thick, low-stress SiN_x_ layer is deposited as the supporting layer of the thermocouples, the contact holes for hot and cold junctions and interconnects are made by RIE (Fig. 2e). All the metal interconnects are sputtered and patterned with 40 nm/100 nm/3000 nm-thick Cr/Pt/Au (Fig. 2f,g) and then covered by plasma-enhanced chemical vapor deposition SiO_2_ with a thickness of 0.5 μm for passivation. Finally, polysilicon is etched in 25% tetramethylammonium hydroxide (TMAH) at 80 °C, and the thermopiles are suspended over insulation cavities due to the wet etching of (111) silicon wafers. Since TMAH etches polysilicon in an isotropic manner and at a faster rate, the etching first proceeds along the vertically etched trenches filled with polysilicon, and then the single-crystalline silicon is removed horizontally. Compared with that using traditional backside etching or etching through release holes, the release time of the large MEMS thermopiles using the MIS process is shortened to ~30 min, achieving better thickness uniformity and avoiding excessive corrosion of the thermocouple lines.

### Characterization of the differential thermopiles

**Fig. 3:**
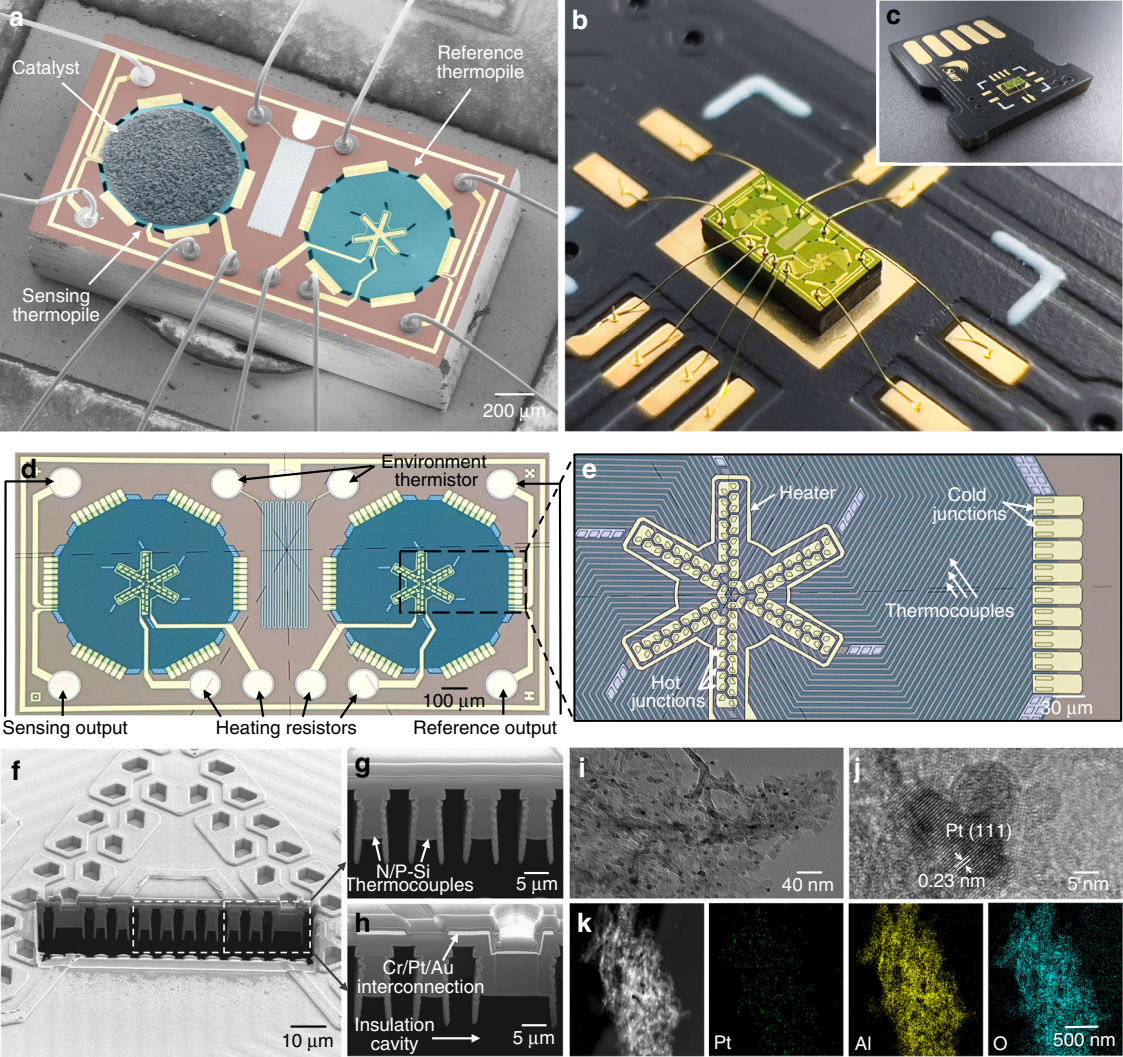
Images of the MEMS differential thermopile-based H_2_ sensor. **a** SEM image of the differential thermopile H_2_ sensor, showing the sensing thermopile loaded with catalyst, while the reference thermopile is left blank. **b**, **c** Optical images of differential thermopiles wire-bonded to a PCB. **d**, **e** Optical images of the differential thermopiles, showing the details of hot junctions, cold junctions, thermocouples, heating resistors, sensing output, and reference output electrodes. **f–h** Cross-sectional view of the suspended thermopiles over the insulation cavity, with magnified views of the thermocouples underneath the supporting SiN_x_ membrane. **i**, **j** TEM images showing the morphology of Pt nanoparticles grown on Al_2_O_3_ nanosheets. **k** EDS characterization showing Pt NPs@Al_2_O_3_ as the catalyst for H_2_ detection

The fabricated MEMS differential thermopile H_2_ sensors are characterized using scanning electron microscopy (SEM) and focused ion beam (FIB) microscopy. The Pt NPs@Al_2_O_3_ catalyst is characterized by transmission electron microscopy (TEM) and element energy dispersive spectroscopy (EDS), as illustrated in Fig. 3. The size of the whole sensor is 1 mm × 2 mm. Each sensor is wire-bonded to a PCB board (Fig. 3b,c). The Pt@Al_2_O_3_ catalyst is uniformly loaded on the sensing region of the sensing thermopile, while the reference thermopile is kept blank. Figure 3d,e shows details of the 54 pairs of thermopiles densely packed within the ~640 µm-diameter suspended SiN_x_ film. The FIB images provide a cross-sectional view of the single-crystalline silicon thermocouples suspended under the SiN_x_ membrane, with a pitch of 3 μm, as shown in Fig. 3f–h. A total of 20 mg of 5 wt% Pt@Al_2_O_3_ catalyst is uniformly dispersed in 1 mL of ethylene glycol and loaded onto the sensing thermopiles. As shown in Fig. 3i–k, Pt nanoparticles are uniformly grown on the Al_2_O_3_ nanosheets shown in the TEM image, with a diameter of 10–20 nm.

### Temperature response of the MEMS differential thermopiles

**Fig. 4:**
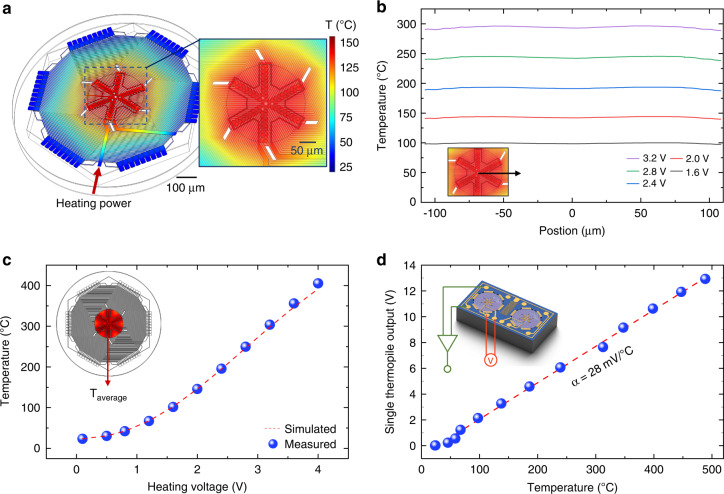
Temperature response of the MEMS differential thermopiles. **a** Finite element modeling of the MEMS thermopiles considering electrical heating, heat transfer in the device, and convection in air. **b** Modeled temperature distribution in the sensing region of the thermopile, showing good uniformity (variation within ±1%). **c** Measured averaged temperature within the sensing region vs. the heating voltage, which is in good agreement with the simulation results. **d** Measured output voltage of a single thermopile vs. the averaged heating temperature within the sensing region, showing a temperature sensitivity of ~28 mV/°C

We first calibrate the temperature response of the MEMS differential thermopiles by heating the sensing region (device center) via the heating resistor. We measure the average temperature within the sensing region using a noncontact infrared thermal imager with a spatial resolution of 20 μm. We also validate the temperature response using finite element modeling (Fig. 4a). The 3D model has the same dimensions as the actual device, including 54 pairs of 4 μm-thick single-crystalline silicon thermocouples arranged in a spiral shape, a 1 μm-thick SiN_x_ suspension film, and a 0.4 μm-thick metal layer for contact at the center and edges of the thermopile. A single-crystalline silicon substrate and a 40 μm-thick air cavity between the thermocouples and the substrate for thermal insulation are also considered in the model for both heat transfer through solid and convection in air. In the simulation, solid heat transfer and convective heat transfer on the upper surface of the device are considered in the heat transfer process. We first evaluate the temperature distribution within the sensing region, which shows a temperature variation of just within ±1% (Fig. 4b). We then calibrate the relationship between the heating voltage and the average temperature of the sensing region, as shown in Fig. 4c, which is in good agreement with the simulation results. The temperature sensitivity of the thermopile is obtained by detecting the output signal as a function of heating temperature, which is measured to be ~28 mV/°C, as shown in Fig. 4d. Given the resistance of a single thermopile of ~540 kΩ and a bandwidth of 200 Hz, we calculate the noise equivalent temperature difference of a single thermopile to be 0.047 mK, allowing us to resolve tiny temperature changes due to the ppm-level H_2_ catalytic reaction in the next experimental section.

### Hydrogen sensing performance of MEMS differential thermopiles

**Fig. 5:**
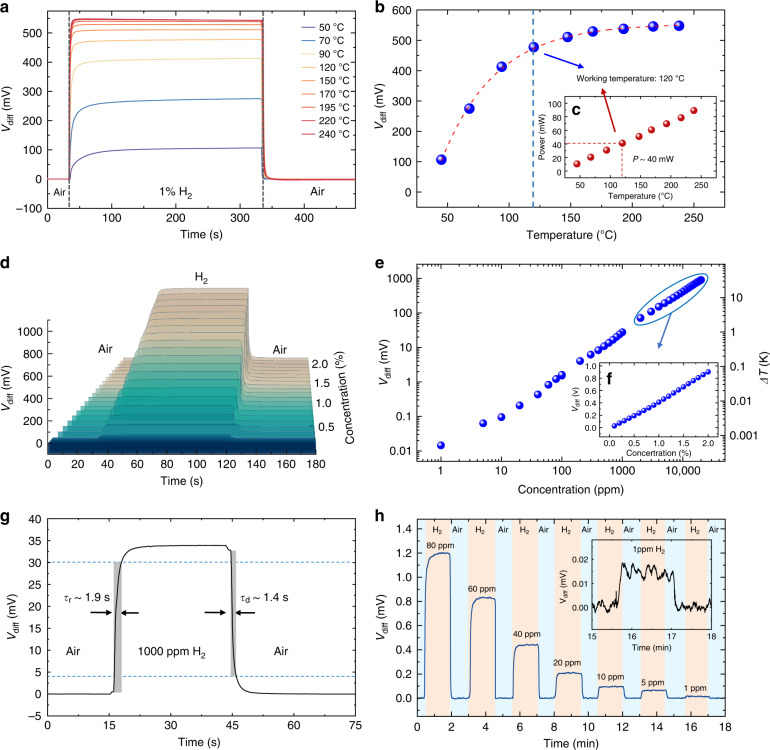
H_2_ sensing performance of MEMS differential thermopiles. **a–c** Sensor response (V_diff_) to 1% H_2_ at different operating temperatures from 50 to 240 °C. An optimized working temperature of 120 °C is determined, with a power consumption of 40 mW. **d–f** Differential output vs. different hydrogen concentrations, showing a linear response within an H_2_ concentration range of 1 ppm to 2%. **g** Measured sensor response and recovery time (t_90_) of 1.9 and 1.4 s, respectively. **h** Zoomed-in view of the sensor response to continuously reduced H_2_ concentration down to 1 ppm

As shown in Fig. 5, we test the H_2_ detection of our MEMS differential thermopiles in air. The sensors are mounted in a test chamber filled with an H_2_/air mixture at a flow rate of 200 sccm. The gas flow is controlled by the high-end-MEMS intelligent gas distribution system with high-precision mass flow meters (MFC), gas mixing units, and gas switching units. The flow control accuracy is calibrated to <1 sccm using an Agilent flowmeter ADM-G6691A. A DC power supply is used to maintain an optimized working temperature. To determine the optimized heating temperature, we switch the supplied gases to the MEMS differential thermopiles between air and a 1% H_2_/air mixture, and the differential output (V_diff_) signals are recorded at operating temperatures from 50 °C to 240 °C, as shown in Fig. 5a. We observe that V_diff_ first linearly increases with the heating temperature in the range of 50–120 °C and then tends to saturate in the range of 120–240 °C. Therefore, we determine an optimized working temperature of 120 °C for the Pt@Al_2_O_3_ catalyst for the H_2_ reaction considering the balanced output signal and power consumption (Fig. 5b, c). In addition, excessive heating can result in larger thermal noise and reduce the catalyst’s lifetime^44^. We thus measure the power consumption of a single thermopile to be ~40 mW. In the subsequent experiments, the operating temperature of the sensors is set to 120 °C.

We then characterize the sensor response (V_diff_) over a range of H_2_ concentrations from 1 ppm to 2%. As shown in Fig. 5d, e, the amplitude of the differential output is linearly proportional to the H_2_ concentration in the range of 1 ppm-2%, with an output of 902 mV at 2% H_2_ and 15 µV at 1 ppm H_2_. It can be inferred that the 15 µV output is caused by a 0.53 mK temperature increase, validating the high-sensitivity design of the differential thermopile H_2_ sensors. These results show that the sensor has excellent detection sensitivity across a concentration range that is more than four orders of magnitude. The response and recovery times (t_90_) when the MEMS differential thermopile sensors are exposed to 0.1% hydrogen are measured to be 1.9 s and 1.4 s, respectively, as shown in Fig. 5g. These values validate the rapid response and recovery of our differential thermopile H_2_ sensors, which are on par with the state-of-the-art of thermoelectric gas sensors. Figure 5h shows magnified sensor response curves when continuously supplying H_2_ gases with reduced concentrations from 80 ppm to 1 ppm, suggesting that our differential thermopile sensors can easily detect 1 ppm H_2_.

**Fig. 6:**
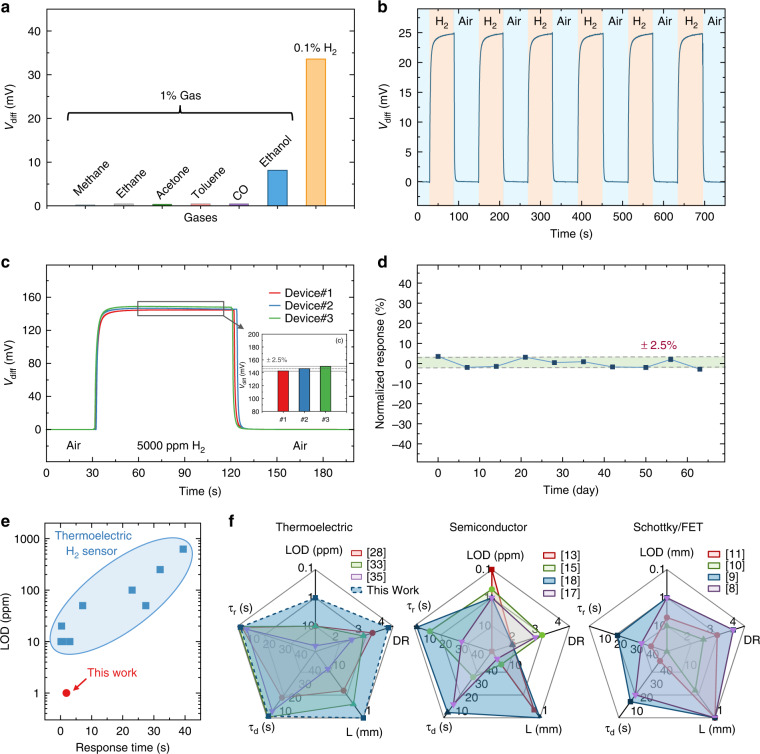
Selectivity, repeatability, uniformity, and stability of the MEMS differential thermopile H_2_ sensors. **a** Measured sensor response to various combustible gases, such as carbon monoxide (1%), methane (1%), ethane (1%), and common VOCs, such as ethanol (1%), acetone (1%), and toluene (1%), compared with the sensor response to 0.1% H_2_. **b** Repetitive testing of the same sensor in 0.1% H_2_. **c** Sensor responses of three randomly selected devices and their responses to the same H_2_ concentration varying only within ±2.5%. **d** Stability of the same sensor every week over 2 months, showing a fluctuation only within ±2.5%. **e**, **f** Comparison of the sensor performance of our MEMS differential thermopiles and other reported H_2_ gas sensors in the literature, where τ_r_ is the response time, τ_d_ is the recovery time, DR is the detection range with the unit of order of magnitude, and L is the feature size of the device

Moreover, we evaluate the selectivity, repeatability, uniformity, and stability of our MEMS differential thermopile H_2_ sensor. To verify the specificity of the sensor to H_2_, the sensors are tested in various combustible gases, such as carbon monoxide (1%), methane (1%), ethane (1%), and common VOCs, such as ethanol (1%), acetone (1%), and toluene (1%). Please note that these results are compared with the sensor response in 0.1% H_2_, as shown in Fig. 6a. Overall, our sensors exhibit excellent selectivity against many combustible gases and VOCs. The sensors are also repeatedly tested at 0.1% H_2_, as shown in Fig. 6b, showing good repeatability. Thanks to the MIS-based MEMS fabrication, the differential thermopile also features good uniformity in terms of the sensor response. We randomly pick 3 sensors and measure their differences in V_diff_ amplitude at 5000 ppm H_2_ varying only within ±2.5%, as shown in Fig. 6c. In addition, we evaluate the sensor stability by keeping a given sensor in an ambient environment and measuring its sensor response (V_diff_) to 1% H_2_ gas every week for over 2 months. It can be inferred from Fig. 6d that the sensor performance is not degraded in ambient conditions and remains stable with a fluctuation within ±2.5%.

Overall, our MEMS differential thermopile H_2_ sensors demonstrate a good detection limit of ~1 ppm, with a fast response and recovery time of only a couple of seconds, across a wide linear detection range from 1 ppm to 2% H_2_ concentration (more than four orders of magnitude). Compared with state-of-the-art thermoelectric or thermopile-based H_2_ sensors^12,23,25–28,32,33,35^, our devices show an order of magnitude better detection limit, an order of magnitude larger detection range, and comparable response and recovery times, as shown in Fig. 6e, f.

More importantly, as shown in Fig. 6f, semiconductor sensors^13,15,17,18^ typically have a better than <1 ppm-level detection limit, while the response time is ~10 s, and the detection range is 2–3 orders of magnitude. Work function-based sensors (e.g., Schottky diode/FET sensors)^8–11^ show a wide linear detection range of up to four orders of magnitude, while the response time is still typically >20 s. In contrast, our MEMS differential thermopile sensors possess outstanding yet balanced device features and sensing performance (in terms of detection limit, detection range, response and recovery time, and device size) compared to their aforementioned counterparts.

## Conclusion

In summary, we design and fabricate a new MEMS differential thermopile H_2_ sensor. The sensor consists of two identical temperature-controlled thermopiles, which detect the temperature change due to the catalytic reaction of H_2_ on the sensing thermopile. By using single-crystalline silicon with a large Seebeck coefficient and high-density thermocouples, the thermopiles exhibit a temperature sensitivity of 28 mV/°C and sub-mK-level temperature resolution. The sensors demonstrate an outstanding yet balanced performance with a detection limit of 1 ppm, a wide linear detection range of 1 ppm-2% (more than four orders of magnitude), and a fast response and recovery time of 1–2 s. Moreover, the sensors also have good selectivity to H_2_, repeatability, and long-term stability. Our MEMS differential thermopile sensors hold promise for trace detection and early warning of H_2_ leakage in a wide range of applications.
